# Enhanced external counterpulsation for management of symptoms associated with long COVID

**DOI:** 10.1016/j.ahjo.2022.100105

**Published:** 2022-02-12

**Authors:** Mohanakrishnan Sathyamoorthy, Monica Verduzco-Gutierrez, Swathi Varanasi, Robyn Ward, John Spertus, Sachin Shah

**Affiliations:** a Department of Internal Medicine, Texas Christian University and University of North Texas Health Science Center School of Medicine, Fort Worth, TX, USA; b Department of Physical Medicine and Rehabilitation, Joe R. and Teresa Lozano Long School of Medicine, San Antonio, TX, USA; c Flow Therapy, Fort Worth, TX, USA; d Texas Cardiovascular Institute, Fort Worth, TX, USA; e Department of Internal Medicine, University of Missouri–Kansas City School of Medicine, Kansas City, MO, USA; f University of the Pacific, Stockton, CA, USA

**Keywords:** Long COVID, EECP, COVID-19, PASC, Long Haul Syndrome

## Abstract

**Study objective:**

Enhanced external counterpulsation (EECP) as a possible therapy for Long COVID.

**Design:**

Retrospective analysis of a contemporary, consecutive patient cohort.

**Setting:**

7 outpatient treatment centers.

**Participants:**

Long COVID patients.

**Intervention:**

15–35 EECP treatments.

**Main outcome measures:**

The change from baseline in 1) Patient Reported Outcome Measurement Information System (PROMIS) Fatigue; 2) Seattle Angina Questionnaire (SAQ); 3) Duke Activity Status Index (DASI); 4) 6-Minute Walk Test (6MWT); 5) Canadian Cardiovascular Society (CCS) Angina Grade; 6) Rose Dyspnea Scale (RDS); and 7) Patient Health Questionnaire (PHQ-9).

**Results:**

Compared to baseline, the PROMIS Fatigue, SAQ, DASI, and 6MWT improved by 4.63 ± 3.42 (p < 0.001), 21.44 ± 16.54 (p < 0.001), 18.08 ± 13.82 (p < 0.001), and 200.00 ± 180.14 (p = 0.002), respectively. CCS and RDS improved in 63% and 44% of patients, respectively. All patients unable to work prior to EECP were able to return post-therapy.

**Conclusions and relevance:**

EECP significantly improved validated fatigue and cardiovascular-related markers in patients with Long COVID.

## Highlights

• This cohort of 16 Long COVID patients treated with EECP showed improvements in validated fatigue and cardiovascular markers.
• After EECP, patients had enhanced quality of life, allowing them to resume normal daily activities and return to work.
• These findings suggest that EECP may be beneficial for the management of Long COVID symptoms and warrants further studies.

## 1 Introduction

Long COVID (also known as post-acute sequelae of COVID-19), involves signs and symptoms that continue or develop after 4 weeks of an acute COVID-19 infection. Cardiopulmonary (chest pain and dyspnea), neuro-psychiatric (fatigue, brain fog, headache, anxiety), and muscular (myalgias) symptoms appear to be the most frequent and concerning in patients with Long COVID [1]. A just published report from Zhang et al. on the one-year health outcomes and symptoms of COVID-19 survivors from Wuhan hospitals reported 45% of patients experiencing at least one symptom consistent with Long COVID [1].

Current approaches are primarily based on self-care or use of a multidisciplinary rehabilitation strategy [2]. Enhanced external counterpulsation (EECP) is a non-invasive outpatient therapy with evidence supporting its use in cardiac and non-cardiac conditions that occur as a result of compromised blood flow/perfusion, vascular inflammation, and/or endothelial dysfunction. Given our recent experience using this modality to improve Long COVID associated fatigue, dyspnea and brain fog, we performed an evaluation of consecutive COVID-19 patients treated with EECP to determine the impact of this modality on Long COVID-associated symptoms [3], [4].

## 2 Methods

This was a retrospective evaluation of a contemporary, consecutive patient cohort of Long COVID patients treated at Flow Therapy treatment centers between April 15, 2021, and September 15, 2021. All patients needed to have a lab documented positive COVID-19 diagnosis prior to March 1, 2021 and referred for the management of Long COVID-related symptoms. The study was approved by the Institutional Review Board at the University of the Pacific. Patients were treated with either 35, 1-h sessions of EECP, or a modified abbreviated regimen of 15 treatments based on previous data [3]. All patients completing the prescribed 15–35 sessions of EECP were included for analysis.

The change from baseline for the following endpoints were evaluated: 1) Patient Reported Outcome Measurement Information System (PROMIS) Fatigue Score; 2) Seattle Angina Questionnaire (summary score (SAQ), angina frequency, physical limitation, and quality of life); 3) Duke Activity Status Index (DASI); 4) 6-Minute Walk Test (6MWT); 5) Canadian Cardiovascular Society (CCS) Angina Grade; 6) Rose Dyspnea Scale (RDS); and 7) Patient Health Questionnaire (PHQ-2 & PHQ-9 when greater than 3 at baseline). Brain fog and ability to return to work were assessed qualitatively. A Wilcoxon Signed Rank test was performed to compare the change from baseline with a Mann-Whitney *U* test used to compare inter-group differences. A Fisher's exact test was utilized to analyze categorical variables. The correlation between baseline symptom severity and the degree of benefit was also assessed.

## 3 Results

A total of 16 patients, (75% female), aged 53.81 ± 15.26 years, were included (Table 1). The approximate time from initial COVID-19 diagnosis to start of EECP therapy was 8.33 ± 3.57 months. Four patients were excluded for not completing EECP therapy and having incomplete data. All patients had Long COVID symptoms, and 9 patients (56%) had concurrent CAD. Seven patients (44%) had hypertension, 7 patients (44%) had dyslipidemia, and 5 patients (31%) had a history of depression or anxiety. There were no documented medication changes over the course of EECP treatment. A total of 7 patients were on beta-blockers, 6 were on aspirin, 4 on statins, 3 on an ACEI/ARB, 3 on coumadin, 3 on an anti-platelet, and 8 on an anti-depressant.

**Table 1:** Change from baseline in functional capacity & anginal markers in long COVID patients after EECP treatment.

Patient (n = 16)	CAD	# EECP Sessions	Age, y	PROMIS-F	SAQ	SAQ-PL	SAQ-AF	SAQ-QOL	DASI	6MWT, feet
1	N	15	21	2	14	42	0	0	5	−80
2	N	15	45	−5	26	17	10	50	14	ND
3	N	15	45	−1	3	0	10	13	11	ND
4	N	15	45	−5	11	33	0	0	36	85
5	N	15	58	−8	45	42	30	63	28	335
6	N	15	64	−2	43	25	30	75	26	130
7	N	25	34	−4	5	17	10	−12	11	ND
8	Y	15	41	−7	13	25	0	13	9	345
9	Y	15	43	−6	17	42	10	0	9	240
10	Y	15	57	−3	−1	8	0	−13	15	85
11	Y	15	69	−7	12	0	10	25	16	28
12	Y	35	62	−5	19	8	0	50	48	0
13	Y	35	66	−3	19	8	10	38	8	440
14	Y	35	66	−12	21	42	10	13	43	520
15	Y	35	66	−3	56	75	30	63	12	200
16	Y	35	79	−7	41	8	40	78	0	20
Mean ± STDEV	N/A	N/A	53.81 ± 15.26	−4.63 ± 3.42	21.44 ± 16.54	24.48 ± 20.30	12.50 ± 12.91	23.80 ± 31.02	18.08 ± 13.82	200.00 ± 180.14
P value	N/A	N/A	N/A	<0.001	<0.001	<0.001	0.001	0.004	<0.001	0.002

Abbreviations: CAD indicates coronary artery disease; EECP, enhanced external counterpulsation; PROMIS-F, Patient Reported Outcome Measurement Information System (PROMIS) Fatigue; SAQ, Seattle Angina Questionnaire summary score; SAQ-PL, Seattle Angina Questionnaire Physical Limitation score; SAQ-AF, Seattle Angina Questionnaire Angina Frequency score; SAQ-QOL, Seattle Angina Questionnaire Quality of Life score; DASI, Duke Activity Status Index; 6MWT, 6-Minute Walk Test; N/A, not applicable; ND, data not available. The threshold for significant differences is p < 0.05.

**Fig. 1:**
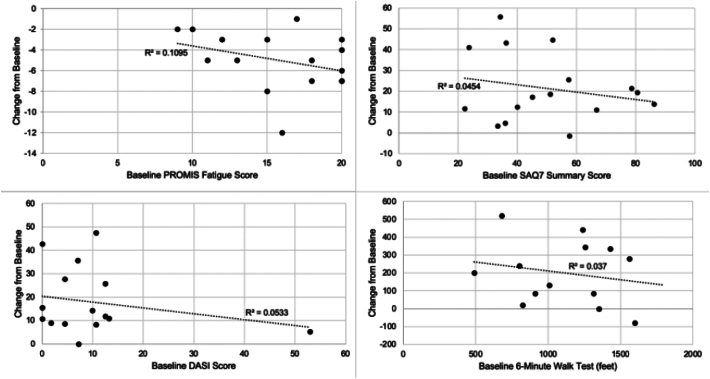
Correlation between baseline symptom severity and degree of improvement. Abbreviations: EECP, enhanced external counterpulsation; PROMIS-F, Patient Reported Outcome Measurement Information System (PROMIS) Fatigue score; SAQ, Seattle Angina Questionnaire summary score; DASI, Duke Activity Status Index; 6MWT, 6-Minute Walk Test.

Compared to baseline, the PROMIS Fatigue score, SAQ, and DASI improved by 4.63 ± 3.42 (p < 0.001), 21.44 ± 16.54 (p < 0.001), and 18.08 ± 13.82 (p < 0.001), respectively. Six-minute walk (6MWT) distance (n = 13) improved significantly by 200.00 ± 180.14 (p = 0.002). Fig. 1 describes the improvement in each patient relative to their baseline. All patients benefited independent of severity of baseline symptom (all R^2^ values <0.110). A comparison of Long COVID patients with and without CAD showed no significant inter-group differences (all p-values >0.156) for PROMIS Fatigue, SAQ, DASI and 6MWT.

Ten patients (63%) reduced their CCS class post-EECP treatment with 25% remaining unchanged (Fig. 2). The RDS improved for 7 patients (44%) and remained the same in 7 patients (44%). Fourteen patients were in CCS class III or IV (marked limitation of ordinary activity or worse) at baseline with only 5 patients in that range post-EECP treatment (88% vs. 31%; p = 0.0032). Ten patients had a baseline RDS of 3–4 (dyspnea when walking at own pace on level ground or worse) compared to 6 post-EECP (63% vs. 38%; p = 0.289) (Fig. 2).

Six patients (38%) reported brain fog at baseline with all reporting an improvement (memory recall, focus, and concentration) by treatment end. For the 2 patients with available PHQ-9 data, both showed notable improvements (13 to 9 and 13 to 2, respectively). Pre-EECP, seven patients did not work or were retired, 2 continued to work despite experiencing Long COVID symptoms, and 6 could not work due to their symptoms (data unknown for 1 person). All 6 patients unable to work previously returned to work/school after EECP therapy.

## 4 Discussion

Since Long COVID is a new disease state, management paradigms are still in evolution. Current clinical approaches world-wide are primarily based on symptom management, nitric oxide-augmenting supplements, self-care, or use of a multidisciplinary rehabilitation. The case of using EECP in patients with Long COVID was first described in April 2021 [3]. Our analysis bolsters that observation and further supports the use of EECP for Long COVID-related symptom management.

In patients with angina, EECP is known to improve markers associated with morbidity and, however, its use in Long COVID was previously considered hypothetical [5].

Long COVID is a disease of endothelial dysfunction and endotheliitis [6]. Shear stress is an important force essential for endothelial homeostasis and normal vascular functioning. Endothelial cells detect shear stress and activate gene expression patterns that result in biochemical alterations to their cellular morphology [7]. Nitric oxide (NO), produced by endothelial nitric oxide synthase (eNOS) in endothelial cells during shear stress, promotes regular blood flow and anti-inflammatory factors. A rise in blood flow velocity and NO enhances endothelial function and overall vascular health [8]. Suppression of endothelial nitric oxide synthase (eNOS) due to reduced shear stress, results in NO deficiency. Reduction in NO is one of the earliest signs of endothelial dysfunction. Therefore, a device (i.e. EECP) capable of delivering shear stress is an attractive method for improving blood flow and vascular tone.

In a sham-controlled trial by Braith et al., EECP demonstrated augmentation of endothelial-derived vasoactive agents (NO, prostaglandin F1 (PGF1), endothelin-1 (ET-1)) and reduction in plasma markers of inflammation (tumor necrosis factor-alpha (TNF-α), high sensitivity C-reactive protein (hs-CRP), monocyte chemoattractant protein-1 (MCP-1), and soluble vascular cell adhesion molecule-1 (sVCAM)) [9].

Cerebral blood flow improvements induced by EECP may explain the benefits in neurological symptoms such as “brain fog”. In ischemic stroke patients receiving external counterpulsation, the mean arterial blood flow velocity in the middle cerebral artery was significantly increased compared to baseline. Further, In post-stroke patients, external counterpulsation improved blood flow in inner carotid arteries and spine arteries by 24% and 14%, respectively [10].

It is important to note that due to the lack of any available data on the use of EECP for Long COVID management without CAD, an initial trial of 15 sessions was utilized as proof-of-concept. As such, a couple patients in the 15 sessions arm needed additional therapy and a duration of treatment from 25 to 35 sessions may ultimately be the most suitable. Future analyses with larger data sets will better assess a dose-response relationship.

In this observational study of 16 Long-COVID patients, EECP significantly improved validated fatigue (PROMIS-F) and cardiovascular-related markers (SAQ, DASI, 6MWT). All affected patients improved symptoms of brain fog and were able to return to work and this study represents the first cohort-based evidence of EECP potentially offering benefit as a therapy to manage Long COVID. Our findings are particularly encouraging as they also include younger patients without underlying CAD, emphasizing physiologic effects that are independent of the epicardial obstructive coronary artery disease. These promising findings are hypothesis generating and should be further explored in a broader clinical investigation.

The following is the supplementary data related to this article.

Fig. 2 Change in CCS and RDS with EECP Treatment. Fig. 2

## Declaration of competing interest

The authors declare the following financial interests/personal relationships which may be considered as potential competing interests: Sachin Shah is a consultant and Swathi Varanasi is an employee of Flow Therapy.

No other competing interests to declare.
