# Supplementary material for: Enhanced external counterpulsation for management of symptoms associated with long COVID
Source: Am Heart J Plus. 2022 Feb 12;13:100105. doi: 10.1016/j.ahjo.2022.100105 (PMC10978164; doi:10.1016/j.ahjo.2022.100105)
Supplement: Fig. 2 — Change in CCS and RDS with EECP Treatment. [file mmc1.docx]

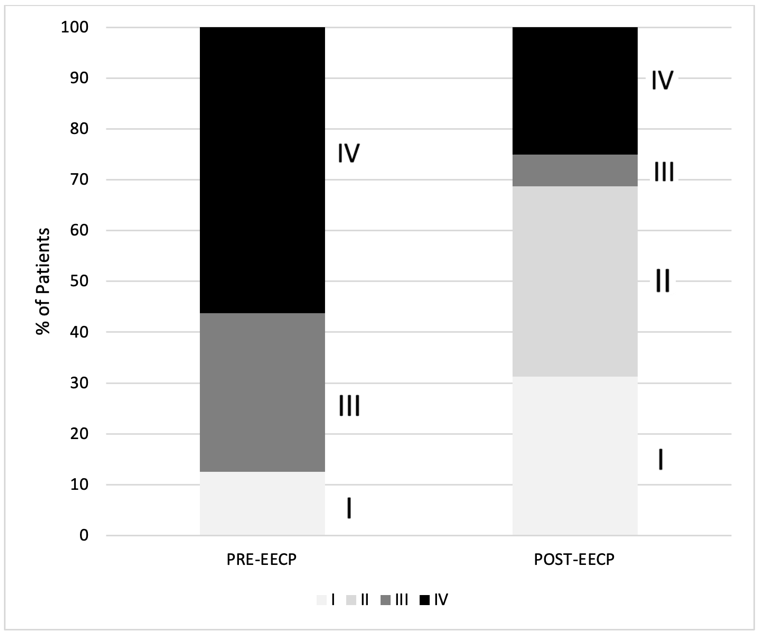
**
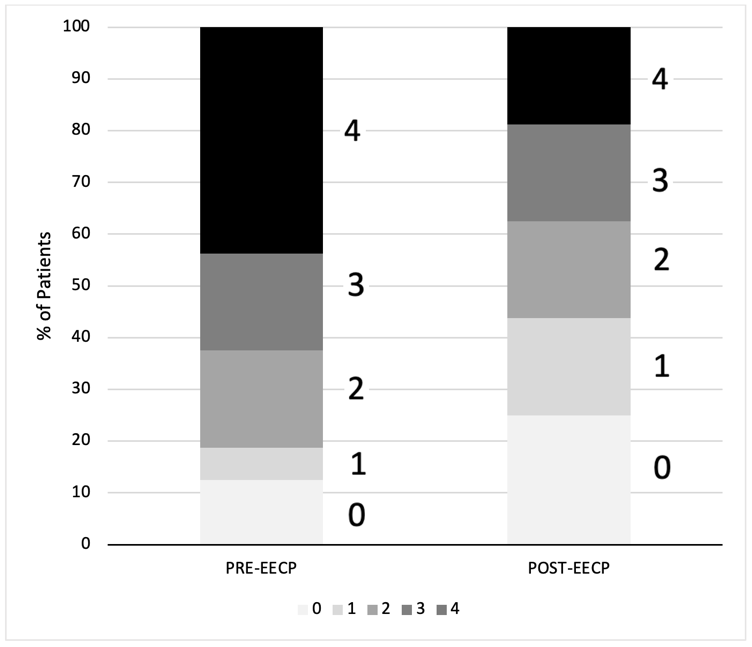
Figure 2. Change in CCS and RDS with EECP Treatment**

Abbreviations: EECP, enhanced external counterpulsation; CCS, Canadian Cardiovascular Society angina grade, RDS, Rose Dyspnea Scale.
